# The quiet cyst: incidental diagnosis and conservative management of the first documented gallbladder ciliated foregut cyst in Palestine: a case report and literature review

**DOI:** 10.1093/jscr/rjaf899

**Published:** 2025-11-14

**Authors:** Ranya Abo Khalaf, Mostafa Ibraheem, Hanin Shatrit, Moayad Nammourah, Orwa Alfallah, Qais Alnjoom

**Affiliations:** Department of Clinical Medical Sciences, Faculty of Medicine and Health Sciences, Palestine Polytechnic University, Hebron P720, State of Palestine; Department of Clinical Medical Sciences, Faculty of Medicine and Health Sciences, Palestine Polytechnic University, Hebron P720, State of Palestine; Diagnostic Radiology Department, Al-Ahli Hospital, Ahli Street, Hebron P720, West Bank, State of Palestine; Diagnostic Radiology Department, Al-Ahli Hospital, Ahli Street, Hebron P720, West Bank, State of Palestine; Diagnostic Radiology Department, Al-Ahli Hospital, Ahli Street, Hebron P720, West Bank, State of Palestine; Department of Clinical Medical Sciences, Faculty of Medicine and Health Sciences, Palestine Polytechnic University, Hebron P720, State of Palestine

**Keywords:** case report, ciliated gallbladder cyst, surgery, developmental anomalies of the gall bladder

## Abstract

Ciliated foregut cysts (CFCs) are rare congenital lesions derived from primitive foregut remnants, typically occurring above the diaphragm; involvement of the gallbladder is extremely uncommon, with only 17 cases reported previously. We present the 18th known case, and the first documented in Palestine, of gallbladder CFC in a 34-year-old female (G5 P4A1) who presented with sudden-onset left flank pain. Imaging revealed mild hydroureteronephrosis due to distal ureteric stones and incidentally identified a well-defined 7 mm cystic structure within the gallbladder fundus on computed tomography, confirmed by ultrasound. The patient had no biliary symptoms, and all other abdominal findings were normal. She was managed conservatively and discharged with outpatient surveillance. Although exceedingly rare, gallbladder CFCs should be considered in the differential diagnosis of incidental gallbladder cystic lesions. Recognition of their imaging features and embryologic origin is essential for accurate diagnosis and appropriate management.

## Introduction

Ciliated foregut cysts (CFCs) are rare congenital lesions derived from remnants of the primitive foregut, which gives rise to the respiratory and upper gastrointestinal tracts. While typically found above the diaphragm, they can rarely appear in subdiaphragmatic organs like the liver and pancreas [1]. Their presence in the gallbladder is extremely rare due to its distinct embryologic origin [2]. To date, only 17 cases have been reported. We present the 18th case worldwide—the first in Palestine and the second in the Middle East [1, 3]. Given their rarity and non-specific imaging features, gallbladder CFCs are usually discovered incidentally. This report aims to improve recognition and support accurate diagnosis and documentation of this unusual lesion.

## Case report

**Figure 1:**
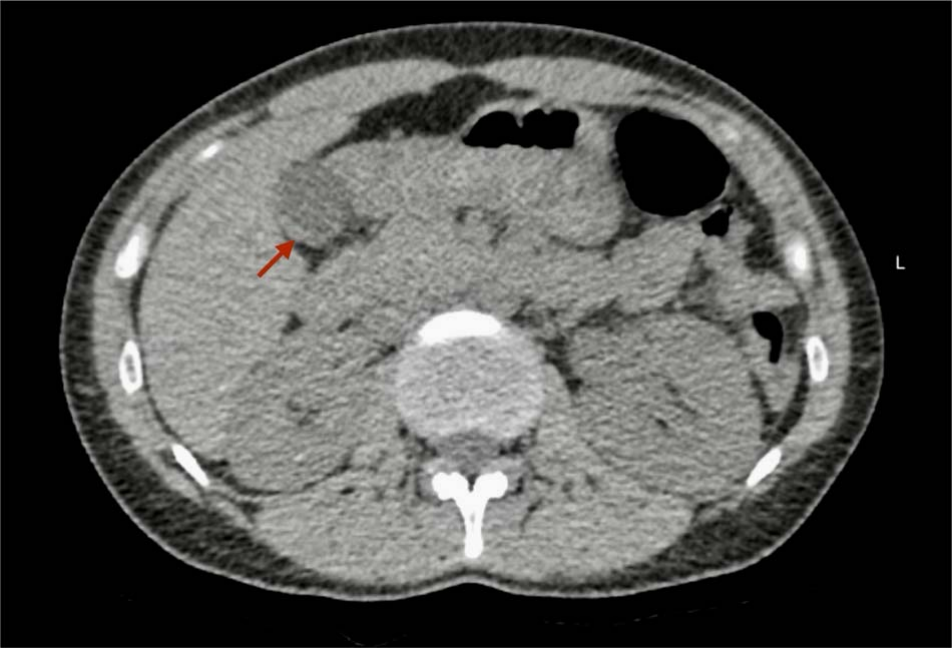
Non-contrast CT scan of the abdomen. The axial plane shows focal thickening of the gallbladder wall (arrow).

**Figure 2:**
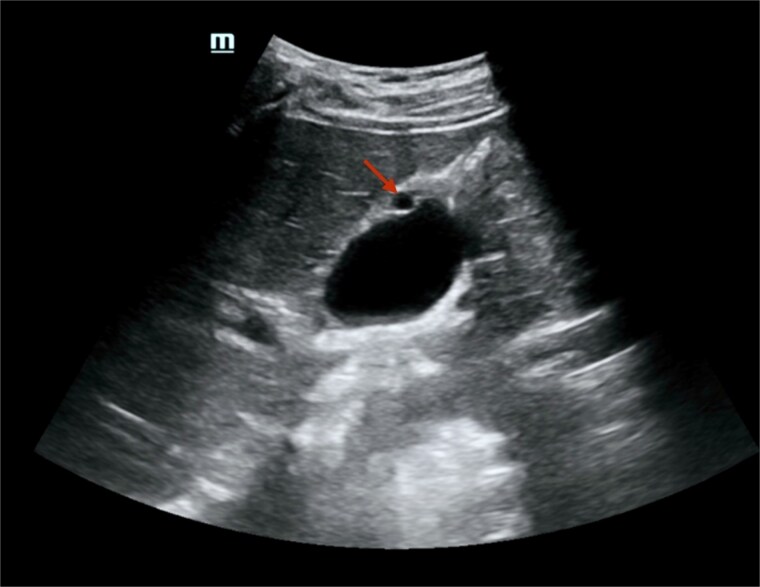
Abdominal ultrasound showed a well-defined, round, anechoic lesion within the gallbladder wall, measuring about 7 mm, likely representing a foregut cyst of the gallbladder. There was no peri-cholecystic edema, obvious shadowing stones, or increased wall thickness.

- Presentation: A 34-year-old multiparous woman (G5 P4 A1) with no history of chronic illness or regular medication use presented to the emergency department with acute left flank pain of 3 h duration. She denied fever, chills, dysuria, hematuria, nausea, or vomiting. On examination, she was alert, oriented, and hemodynamically stable, with localized tenderness over the left flank. Cardiopulmonary, neurological, and musculoskeletal examinations were unremarkable. Laboratory investigations—including complete blood count, urinalysis, and serum creatinine—were within normal ranges.
- Imaging findings: A non-contrast computed tomography (CT) scan of the abdomen revealed a mild left hydroureteronephrosis caused by a distal obstructive ureteric stone. Incidentally, the scan showed a focal thickening of the gallbladder fundus wall (Fig. 1). Correlation with abdominal ultrasound identified a well-defined 7 mm cystic lesion within the gallbladder wall (Fig. 2). The liver, spleen, and other abdominal organs appeared normal on CT.
- Management: The patient was managed conservatively with intravenous fluids and analgesia during hospitalization.
- Follow-up: She was discharged with advice for oral hydration and outpatient follow-up for both the ureteric stone and the incidental gallbladder lesion.

## Discussion

Cystic lesions of the gallbladder are uncommon and may be congenital, acquired, or neoplastic. Among congenital types, ciliated cysts are benign lesions believed to originate from the anterior primitive gut [4]. During early embryogenesis, the primitive foregut forms in the third–fourth week of gestation and subsequently gives rise to a diverse set of endodermal derivatives. The ventral foregut develops into the pharynx, esophagus, stomach, proximal duodenum, as well as the hepatobiliary system (including the liver, intra/extrahepatic bile ducts, and gallbladder) and the ventral pancreatic bud. In contrast, the dorsal foregut contributes to the development of the respiratory tract and thyroid gland. Aberrant sequestration of epithelial rests from this developmental process is thought to underlie the formation of foregut cysts. The characteristic lining of ciliated pseudostratified columnar epithelium with underlying smooth muscle in these cysts reflects their embryonic origin and distinguishes them from true biliary epithelium [5].

These cysts are typically unilocular, lined by pseudostratified, ciliated, mucin-secreting columnar epithelium [6]. While they most commonly occur above the diaphragm, such as in the bronchial tree, esophagus, or mediastinum [3], they have also been reported below the diaphragm, particularly in the liver as ciliated hepatic foregut cysts, with over 100 documented cases. In contrast, gallbladder CFCs are extremely rare [7] and are thought to result from aberrant embryologic differentiation, leading to ectopic ciliated epithelium in sites like the liver, gallbladder, or pancreas [8].

According to Table 1, from 1995 to 2025, 18 cases of gallbladder CFCs were reported, and 1 case was excluded in our review table and analysis because it was different from the rest, as it was in the liver with some gallbladder involvement. So, our review showed a strong female predominance (78%). Most patients (14 cases) were symptomatic, predominantly with right upper quadrant pain, while 4 cases were detected incidentally. The neck of the gallbladder was the most frequent location (61%), followed by the body, fundus, and Calot’s triangle [9], possibly reflecting an embryologic tendency related to nearby biliary structures.

**Table 1:** Reported cases of cysts in the gallbladder worldwide (1995–2025)

Author	Year	Sex	Age	Symptoms	Location	Size	Locularity	Content	Imaging	Therapy
Kakisubata	1995	M	71	Asymptomatic	Body	Unspecified	Unilocular	Unspecified	Ultrasound	Open
Nam	2000	F	36	Asymptomatic	Fundus	15 mm	Unilocular	Mucus	US, CT	Laparoscopy
Hirono	2002	F	43	Asymptomatic	Neck	25 mm	Unilocular	Mucus	US, CT, MRI	Open
Muraoka	2003	F	37	Asymptomatic	Body	24 mm	Unilocular	Mucus	US, CT with contrast	Open
Bulut	2010	F	41	RUQ pain	Neck	35 mm	Unilocular	Mucus	Unspecified	Laparoscopy
Tunçyürek S	2013	F	42	RUQ pain	Body	7 mm	Unilocular	Mucus	US	Laparoscopy
Giakoumidis	2014	F	29	Epigastric pain	Neck	30 mm	Unilocular	Mucus	US, MRI (adjacent to gallbladder)	Laparoscopy
Hwang	2015	F	39	RUQ pain	Neck	35 mm	Unilocular	Mucus	US, CT	Laparoscopy
Lee	2015	M	61	RUQ pain	Body	27 mm	Unilocular	Gelatinous	US, CT	Laparoscopy
Han	2016	F	20	RUQ pain	Neck	16 mm	Unilocular	Mucus	US/CT (not described)	Unspecified
Agarwal	2016	M	9	RUQ pain, vomiting	Neck	30 mm	Unilocular	Unspecified	US, MRI (adjacent to gallbladder)	Laparoscopy
Farrugia	2017	M	72	RUQ pain, nausea	Neck	45 mm	Unilocular	Unspecified	CT (adjacent to gallbladder)	Open
Wissem	2017	F	34	RUQ pain	Neck	30 mm	Unilocular	Mucus	US, CT with contrast	Open
Chikanori	2021	F	50	Asymptomatic	Neck	17 mm	Unilocular	Mucus	US, CT with contrast	Open
Baig and Priya	2022	F	20	RUQ pain	Calot’s Triangle	50 mm	Unilocular	Mucus	US, CT with contrast	Laparoscopy
Ghanghoria *et al*.	2023	F	30	RUQ pain, vomiting	Neck	25 mm	Unilocular	Mucus	US, MRCP	Laparoscopy
Present case	2025	F	34	Asymptomatic	Fundus	7 mm	Unilocular	Unspecified	US, CT scan	Conservative

Lesion sizes ranged from 7 to 50 mm. The smallest (7 mm) appeared in both the current 2025 case (fundus) and a 2013 report (body). The largest, a 50 mm cyst in Calot’s triangle, was documented in 2022. Patient ages were from 9 to 72 years, with a median of 36.5 years. Over half of the cases occurred in individuals aged 30–45, indicating a diagnostic concentration in mid-adulthood.

The imaging characteristics of our case a small-sized cystic lesion with clearly defined margins, and fundic location which suggest a CFC, a rare congenital lesion originating from embryonic foregut remnants. Although asymptomatic, its rarity and developmental origin justify ongoing monitoring, especially given the few cases reported globally.

The differential diagnosis of gallbladder cystic lesions is broad and includes congenital, acquired, and neoplastic entities. Congenital lesions encompass simple epithelial cysts, duplication cysts, mesothelial cysts, dermoid cysts, and CFCs, all of which are rare and often discovered incidentally [10]. Acquired cystic changes may result from dilated Rokitansky–Aschoff sinuses in adenomyomatosis, retention cysts secondary to obstruction, or inflammatory pseudocysts related to chronic cholecystitis [10]. Neoplastic cystic lesions include cystadenomas and cystadenocarcinomas, which may mimic benign cysts radiologically but carry malignant potential [11]. Other tumor-like conditions such as cholesterol polyps, xanthogranulomatous cholecystitis, and heterotopic tissue (gastric or pancreatic) should also be considered [12]. Distinguishing CFCs from these entities relies on careful correlation of imaging with histopathology, as the presence of pseudostratified ciliated columnar epithelium with smooth muscle wall is pathognomonic. Recognition of these features is crucial to avoid misdiagnosis and unnecessary surgical intervention, particularly in asymptomatic patients.

As a conclusion, CFCs of the gallbladder represent infrequent congenital anomalies, with limited cases described in the literature. This report presents the smallest documented lesion to date and the first case reported in Palestine. Its incidental discovery highlights the importance of considering such cysts in the differential diagnosis of gallbladder lesions. Thorough documentation contributes to improved recognition, enhances clinical awareness, and supports better-informed management of these uncommon entities.

## Data Availability

All data supporting the findings of this case report are included within the article. Other details or clarifications are available from the corresponding author upon reasonable request.
